# Traversing the bench to bedside journey for iNKT cell therapies

**DOI:** 10.3389/fimmu.2024.1436968

**Published:** 2024-08-07

**Authors:** Julie O’Neal, Melissa Mavers, Reyka G. Jayasinghe, John F. DiPersio

**Affiliations:** ^1^ Division of Oncology, Department of Medicine, Washington University School of Medicine, Saint Louis, MO, United States; ^2^ Siteman Cancer Center, Washington University in St. Louis, St. Louis, MO, United States; ^3^ Division of Hematology and Oncology, Department of Pediatrics, Washington University School of Medicine, St. Louis, MO, United States

**Keywords:** invariant natural killer T cell, iNKT, chimeric antigen receptor, CAR, graft versus host disease, GVHD, immune cells, clinical trial

## Abstract

Invariant natural killer T (iNKT) cells are immune cells that harness properties of both the innate and adaptive immune system and exert multiple functions critical for the control of various diseases. Prevention of graft-versus-host disease (GVHD) by iNKT cells has been demonstrated in mouse models and in correlative human studies in which high iNKT cell content in the donor graft is associated with reduced GVHD in the setting of allogeneic hematopoietic stem cell transplants. This suggests that approaches to increase the number of iNKT cells in the setting of an allogeneic transplant may reduce GVHD. iNKT cells can also induce cytolysis of tumor cells, and murine experiments demonstrate that activating iNKT cells *in vivo* or treating mice with *ex vivo* expanded iNKT cells can reduce tumor burden. More recently, research has focused on testing anti-tumor efficacy of iNKT cells genetically modified to express a chimeric antigen receptor (CAR) protein (CAR-iNKT) cells to enhance iNKT cell tumor killing. Further, several of these approaches are now being tested in clinical trials, with strong safety signals demonstrated, though efficacy remains to be established following these early phase clinical trials. Here we review the progress in the field relating to role of iNKT cells in GVHD prevention and anti- cancer efficacy. Although the iNKT field is progressing at an exciting rate, there is much to learn regarding iNKT cell subset immunophenotype and functional relationships, optimal ex vivo expansion approaches, ideal treatment protocols, need for cytokine support, and rejection risk of iNKT cells in the allogeneic setting.

## Introduction

Invariant natural killer T (iNKT) cells are immune cells with both innate and adaptive properties that play key roles in immune responses to both pathogens and malignant cells. These cells are named for their expression of a semi-invariant αβ T cell receptor (TCR) and variable expression of surface proteins similar to natural killer (NK) cells along with functional properties similar to both populations. Murine iNKT cells express a Vα14-Jα18 alpha chain (1) which pairs with Vβ8.2, Vβ2 or Vβ7. Human iNKT cells express a Vα24-Jα18 (1) TCR α chain that pairs with a Vβ11 β chain. The semi-invariant TCR is restricted to the monomorphic major histocompatibility complex (MHC) class I-like molecule, CD1d (2), which heterodimerizes with β-2 microglobulin (β2M) and, unlike typical MHC complexes, presents glycolipids rather than protein peptides (3). The glycolipid α-galactosylceramide (αGalCer), originally isolated from a marine sponge (4) is the most well-known glycolipid that when complexed to CD1d induces iNKT cell activation (4–6). Early studies of murine iNKT cells showed that they are CD4^+^CD8^-^ or CD4^-^CD8^-^ (double negative (DN) (5, 6). Human iNKT cells isolated from PBMC, similar to mice, are mostly CD4^+^CD8^-^ or DN, although a CD4^-^CD8^+^ population has also been described (7, 8). Both CD4^+^ and DN iNKT cells induce T-helper 1 cells (Th1) cytokines interferon gamma (IFN-γ) and tumor necrosis factor (TNF) upon activation, while CD4^+^ cells were found to also express Th2 like cytokines interleukin-4 (IL-4), and IL-13 (8, 9). Further work has described three main transcriptionally and functionally distinct subsets in mice: iNKT1, iNKT2 and iNKT17. iNKT1 are Th1-like, express T-bet, produce primarily interferon gamma (IFNγ), and are predominantly DN, though can also be CD4^+^; iNKT2 are Th2-like, express high levels of promyelocytic leukemia zinc finger (PLZF), primarily produce interleukin IL-4, and are CD4^+^; while Th17-like iNKT17 cells express RAR-related orphan receptor-gamma (RORγt), secrete primarily IL-17, and are DN (10–21). These patterns of cytokine production and CD4 expression (or lack thereof) correlate with the results from human studies of CD4^+^ and DN iNKT cells noted above. Importantly, functional distinctions for the iNKT1, iNKT2 and iNKT17 subsets have been demonstrated, such as maximal cytotoxic function in iNKT1 cells and graft-versus-host disease (GVHD) suppressive capacity in iNKT2 and iNKT17 cells (15–23). Additionally, in both mice and humans, growing evidence suggests the existence of a subset of NKT cells characterized by IL-10 secretion and immunosuppressive function (NKT10) (24–27). These cells have been detected in adipose tissue and the intestinal compartment, though whether they are present in peripheral blood remains unclear (25–28). Although data is lacking, it might be worth investigating the potential ability of NKT10 cells to block GVHD and also the negative effect NKT10 cells may have on the anti-tumor function of chimeric antigen receptor (CAR-iNKT) or iNKT cells. Many current research efforts remain focused on better understanding the relationship between immunophenotype and function of iNKT cells, particularly in the context of anti-GVHD and cytotoxic activity against malignant cells (whether unedited or engineered to express a CAR). Given their multifunctionality, there is significant interest in the potential of iNKT cells as cellular therapies. This review will focus on the progress in harnessing iNKT cells for GVHD suppression and targeting malignant disease.

## iNKT cells for GVHD suppression

### Murine iNKT cells suppress GVHD

Substantial preclinical data demonstrates an ability of iNKT cells to suppress GVHD in the context of hematopoietic stem cell transplantation (HSCT) as we previously reviewed (29). Use of a total lymphoid irradiation (TLI) and antithymocyte globulin (ATG) conditioning regimen for HSCT led to relative preservation of host iNKT cells and was associated with protection from GVHD in murine models (30–32). *In vivo* activation of iNKT cells using αGalCer, a glycolipid antigen known to activate the semi-invariant TCR (4), also led to prevention of murine GVHD that was dependent on CD1d, regulatory T cells (Tregs), IL-4, and STAT6 (33–37). This approach was also shown to potentiate the effects of other GVHD-prevention approaches, such as post-transplant cyclophosphamide in a murine haploidentical model of HSCT (38). The adoptive transfer of even very low numbers of murine iNKT cells, whether donor or third-party derived, prevented acute GVHD in an MHC-mismatched setting without other chemoprophylaxis (and without loss of the graft-versus-leukemia (GVL) effect) through *in vivo* expansion of Tregs (10, 12–14). Importantly, this function was restricted to CD4^+^ iNKT cells or to iNKT2 and iNKT17 cells (11, 13). The adoptive transfer of murine iNKT cells has also been shown to suppress active chronic GVHD (33).

### Human iNKT cells suppress GVHD in pre-clinical studies

The aforementioned studies naturally led to efforts to demonstrate that human iNKT cells could suppress GVHD. This is first supported by data demonstrating that increased iNKT cell number in HSCT grafts correlated with reduced GVHD in patients (39, 40). Further, multiple studies show GVHD protective function of human iNKT cells in a xenogeneic mouse model whereby infusion of human peripheral blood mononuclear cells (PBMC) elicits GVHD in immunocompromised NSG mice (41–43). Bulk human iNKT cells adoptively transferred into the xenogeneic GVHD model led to improvement in survival, weight loss, and histopathologic evidence of disease (43). To elucidate immunophenotypically defined iNKT subsets that play a role in GVHD, CD4^+^ and CD4^-^ human iNKT cells were sorted and adoptively transferred separately into the xenogeneic GVHD model (41). Only CD4^-^ iNKT cells could suppress xenogeneic GVHD, with an *in vivo* reduction in both the percent of murine CD11c+ splenic dendritic cells (DC) and the activation of human T cells. In another study, human cord blood (CB)-derived hematopoietic stem cells (HSC), of interest due to their massive expansion capacity, were engineered to express the invariant TCR, differentiated *ex vivo*, and shown to suppress xenogeneic GVHD without loss of the GVL effect (42). The engineered iNKTs were all CD4^-^CD8^-/+^, consistent with the data above that CD4^-^ iNKT cells block GVHD. The exact mechanism(s) by which human iNKT cells suppress xenogeneic GVHD remains to be determined. Bulk iNKT cells, as well as individual CD4^+^ and CD4^-^ populations were shown to directly reduce T cell activation by CD3/28 beads *in vitro* (43, 44), while both CD4^-^ and, to a lesser extent, CD4^+^ iNKT cells had cytotoxic function towards murine and human DC *in vitro* (41). HSC-engineered iNKT cells were also shown to kill human CD14^+^ cells (42). Of interest, human iNKT cells were also shown to preferentially induce apoptosis of conventional DC *in vitro*, reducing their ability to activate T cells in an MLR and skewing the DC population towards more tolerogenic plasmacytoid DC (45). Together, multiple mechanisms likely contribute to inhibition of GVHD by iNKT cells, however, due to the discrepancies in murine and xenogeneic studies, there is not yet consensus on whether CD4 is a reliable marker for the GVHD suppressive function of iNKT cells.

### Clinical trials harnessing iNKT cells for GVHD suppression

These impressive preclinical results led to clinical trials assessing the power of iNKT cells to prevent GVHD. The aforementioned TLI/ATG conditioning has proven effective in clinical trials and is now in use for some patients (46–49). *In vivo* administration of αGalCer to activate iNKT cells is being tested in clinical trials, with initial results demonstrating safety (50). Recent Phase 2 results showed a significant reduction in acute GVHD and improvement in survival when six weekly infusions were combined with calcineurin inhibitor/methotrexate-based GVHD prophylaxis (NCT04014790) (51);. Another intriguing approach is graft manipulation. Building on the success of Orca-T, a graft engineering approach which optimizes the T:Treg cell ratio and timing of administration in the HSCT process (52), the newest Orca product, Orca-Q, additionally adds back unmanipulated donor iNKT cells with the goal of further enhancing GVHD prevention in patients undergoing haploidentical stem cell transplantation and is now enrolling (NCT03802695) (53, 54). However, to date no trials employing the selective adoptive transfer of human iNKT cells have been registered, though one listed trial purports to identify the ideal GMP-compliant expansion methods to facilitate this (NCT03605953).

### Challenges in clinical development of iNKT cell-based approaches to GVHD suppression

One challenge with translating the adoptive transfer (or *in vivo* activation) of iNKT cells for GVHD prevention or treatment into clinical use is the conflicting murine and human/xenograft preclinical data. It remains unclear whether CD4^+^ iNKT cells/iNKT2 cells or CD4^-^ cells are best at this function. Additionally, multiple mechanisms are proposed including direct suppression of donor T cell activation and killing of antigen presenting cells (APC) of host or donor origin that could present host antigen to donor T cells. The possibility remains that *both* populations have GVHD-suppressive capacity through different mechanisms of action and could potentially have an additive or synergistic effect if bulk iNKT cells are used. Given the drawbacks of the xenogeneic model, such as the influence of murine APC, murine glycolipid and peptide antigens, and murine cytokines and chemokines on adoptively transferred human iNKT cells, one could suggest that the purely murine model results are more reliable. Yet, given clear inherent differences between murine and human iNKT cells, as well as the correlative human studies suggesting that donor graft CD4^-^ iNKT cells are most protective against GVHD, one might weigh the xenograft results heavier. Further, the data does not yet fully address the impact of specific iNKT subsets on relapse rates, as murine studies show that CD4^+^ iNKT cell-mediated GVHD suppression does not result in loss of GVL, while both murine and human preclinical studies suggest malignant cell killing function lies with CD4^-^/iNKT1 which could potentially allow for synergy with T cell-mediated malignant cell killing (11, 44). These challenges highlight the need for carefully designed clinical trials to provide answers.

## iNKT cells for targeting malignant disease

### Anti-tumor effects of unedited iNKT cells in mice and humans

Many *in vivo* murine tumor models demonstrated anti-tumor activity of iNKT cells by *in vivo* treatment with IL-12, αGalCer, αGalCer+ IL-12, αGalCer pulsed dendritic cells, or adoptive transfer of iNKT cells and others (mouse studies extensively reviewed in (55)). Data showing that human cancer patients with higher iNKT cell numbers at tumor (56, 57) and/or in PB (58) have better outcomes support an anti-tumor role for human iNKT cells. Accordingly, reduced iNKT cell numbers and detection of functionally impaired iNKT cells associate with worse outcomes (59, 60). iNKT cells induce tumor cytolysis via multiple mechanisms (55). Activated iNKT cells can directly kill CD1d^+^ tumor cells via their semi-invariant TCR through Fas/FasL interactions and secretion of cytotoxic granules (61–64). They can also recruit other immune cells to facilitate tumor cell lysis. Interaction of iNKT with immature DC’s leads to DC CD40 and iNKT CD40L engagement, DC maturation, and secretion of IL-12. IL-12 further activates iNKT to secrete IFNγ which can activate multiple cell types such as NK (65) and CD8^+^ T-cells to mediate tumor killing (61, 66, 67). They can also affect the tumor suppressive microenvironment by directly killing CD1d^+^ tumor associated macrophages (TAM) (68, 69).

### Clinical trials testing human iNKT cells for anti-tumor function

Several clinical trials tested whether treatment with αGalCer alone, αGalCer pulsed CD1d^+^ APC, or other CD1d^+^ cell types (monocyte derived DC (moDC), mature DC, immature DC rich APCs) would activate and expand iNKT cells *in vivo* in patients in a variety of cancer types (reviewed in (55, 70, 71)). Most of these early phase studies demonstrated safety including in patients who received four doses of 1x10^9^ moDC (72). An ongoing study is testing co-delivery of nanoparticle encapsulated IMM60 (iNKT activator) and NY-ESO-1 peptides (NCT04751786). In many studies, increases in iNKT cell numbers and IFNγ levels were observed but unfortunately, unlike many pre-clinical mouse studies, limited anti-tumor efficacy was seen and ranged from no response up to a partial response (PR).

A number of clinical trials have tested the safety and efficacy of the adoptive transfer of *ex vivo* expanded iNKT cells in patients with various cancer types. Although there were differences across studies including *ex vivo* production protocols, purity, and dose, infusion of autologous unmodified iNKT cells alone (73) or combined with DC and CD8^+^T cells (74), IL-2 (75), GM-CSF (76), or an immune checkpoint inhibitor (77) were all importantly, found to be safe (doses up to 1x10^10^/m^2^). Unfortunately, the efficacy in these studies was mostly limited. However, in a Phase 2 randomized clinical trial, HCC patients treated with *ex vivo* expanded iNKT cells and trans arterial embolization (TAE) had longer progression free survival (PFS) and overall survival (OS) than patients treated with TAE alone. Five TAE-iNKT patients and one TAE patient had complete responses (CR) (78). Ongoing trials are testing efficacy of autologous iNKT cell infusions (NCT02562963) or an allogeneic ‘off the shelf’ iNKT cell product (agenT-797; MiNK Therapeutics) without lymphodepleting chemotherapy plus a multi-drug combination (NCT06251973). The safety and efficacy outcomes from these and subsequent trials testing new approaches to enhance iNKT anti-tumor responses will be of clinical interest to the iNKT field.

### Advantages of iNKT cells as a platform for CAR therapy

To date, unmodified iNKT cell therapy has not generated sustained complete responses (CR) in human cancer patients. Genetic modification of iNKT cells to express chimeric antigen receptor (CAR) proteins (CAR-iNKT) may maximize the anti-tumor function of iNKT cells. Further, as discussed above, iNKT cells do not cause, and in fact, prevent GVHD, making them a natural “off-the-shelf” cell source. This is in direct contrast to T cells, which require genetic deletion of the TCR to prevent GVHD, which may negatively impact the product *in vivo* (79). Allogeneic CAR-iNKT therapy addresses multiple limitations of autologous CAR-T cell therapy including production failures, limited *in vivo* expansion and durability of CAR-T response likely due to poor health and/or immunophenotype of T cells in heavily pre-treated patients, high cost, complicated logistics, and lengthy time to treatment. Potentially fatal cytokine release syndrome (CRS) and immune effector cell-associated neurotoxicity also occur with CAR-T cell therapy (80). Using an *in vitro* CRS model, we found CAR-iNKT cell therapy may have a lower propensity for, or reduced intensity, of CRS (62). In two ongoing clinical trials, only grade 1 or 2 CRS has been observed to date (discussed below) (81, 82); further preclinical studies and clinical trials will elucidate this aspect of CAR-iNKT cell safety.

### Preclinical studies of CAR-iNKT cells

CAR-iNKT cell therapy is a promising, but relatively young field, with the first publication in 2014 (83). *In vitro* and *in vivo* anti-tumor efficacy of CAR-iNKT cells has been demonstrated across many malignancies, with targets including CD19 for B-cell malignancies (62, 64, 84–86), BCMA (62, 63) or CD38 (63) for multiple myeloma, GD2 for neuroblastoma (87), TCRVβ for T-cell lymphoma (88), and B7H3 for multiple solid tumors (89). Unlike CAR-T cell therapy, where tumor cell killing is mediated solely by the CAR, an advantage of CAR-iNKT cells is they can kill via the CAR and their TCR. Engagement of the endogenous invariant TCR on iNKT cells can lead to killing of CD1d expressing tumor cells in the presence of αGalCer with or without co-expression of CAR target (61–64, 87), which could be relevant for CD1d expressing tumors including early stage myeloma (90), some B cell malignancies, marginal zone leukemia, mantle cell leukemia (64) and glioblastomas (91). Although these *in vitro* studies included addition of αGalCer, the iNKT cell TCR likely also interacts with CD1d loaded with endogenous tumor lipids (92). This dual targeting may improve efficacy of CAR-iNKT cells and potentially prevent antigen escape, which occurs in single targeted CAR-T cell therapy targeting CD19, BCMA and GPRC5D (93, 94).

A small set of preclinical studies compared *in vivo* efficacy of CAR-T cell and CAR-iNKT cell therapies. Of note, the therapeutic dose of CAR-iNKT cells in most preclinical studies is typically higher (5-10x10^6^/mouse) than CAR-T cell doses (0.5-2x10^6^/mouse), suggesting that higher doses or multiple dosing may be needed for clinical use. However, two studies directly comparing the efficacy of CAR-T cells and CAR-iNKT cells using tumor co-expressing CAR target and CD1d suggests this may not be the case. Dosed matched CD19 CAR-iNKT cells had superior efficacy to CD19 CAR-T cells in an NSG/CD19^+^CD1d^+^ Ramos mouse model (64). Dose matched BCMA CAR-iNKT cells also had better anti-myeloma efficacy than BCMA CAR-T cells in an NSG/BCMA^+^CD1d^+^MM.1S mouse model (95). In our study, however, dose-matched BCMA CAR-iNKT cells were inferior to CAR-T cells in an NSG MM.1S myeloma mouse model with CD1d^-^ tumor cells (62). This suggests that CAR-iNKT cells may be better suited than CAR-T for tumor cell types proven to express CD1d and provide rationale for treating patients with tumors positive for both the CAR target and CD1d with CAR-iNKT cells. To further address this pre-clinically, side by side efficacy studies comparing outcome of tumor bearing mice with either CD1d^+^ or CD1d^-^ tumor cells and treated with CAR-iNKT or CAR-T cells are needed. In separate studies, it was shown that GD2 CAR-iNKT cells demonstrated better tumor trafficking than GD2 CAR-T cells (83); these results and poor efficacy of GD2 CAR-T cells in human trials provided key rationale for an ongoing trial testing GD2 CAR iNKT cells in neuroblastoma patients discussed below.

It is important to note that the above studies used human effector and tumor cells in NSG immunodeficient mouse xenograft models. Using allogeneic fully murine mouse models, Simonetta et al. (67) demonstrated superior dose-matched CD19 CAR-iNKT cell efficacy compared to CAR-T cells in the presence of recipient immune cells using an allogeneic tumor mouse model. This was shown to be due to cross priming of host CD8^+^ T cells which led to long lasting anti-tumor immunity. This study highlights the importance of careful consideration of preparatory regimens prior to CAR-iNKT cell infusions in clinical trials and requires further preclinical study.

T cells engineered to express a CAR construct with a CD28 costimulatory domain have been clearly shown to demonstrate quick cytotoxicity kinetics, which could be good for aggressive disease, yet are less durable than 4-1BB containing CAR-T cells which have slower killing kinetics and longer persistence, thought to be appropriate for less aggressive disease (96, 97). However, co-stimulatory domains are less well studied and optimized for CAR-iNKT cells. *In vitro* studies of CD28, 4-1BB or CD28/4-1BB containing CAR-iNKT cells showed similar killing activity, and of those, higher Th1 cytokine secretion was seen in CAR-iNKT cells containing a 4-1BB co-stimulatory domain (63, 83). Heczey et al. (83) also showed similar survival of tumor engrafted mice treated with CD28, 4-1BB, or CD28/4-1BB containing CAR-iNKT cells, and best *in vivo* persistence of CD28/4-1BB CAR-iNKT cells. Future studies will continue to discover best CAR designs for iNKT cells.

As discussed above, there is data suggesting that CD4^+^ and CD4^-^ iNKT cells have some distinct phenotypes and functions (7–9). A 2011 study (98) characterized expanded and activated iNKT cells from PBMC and showed the CD4^+^ and DN phenotypes and function were generally consistent with the prior studies, while CD8α^+^ iNKT cells had the highest cytotoxicity towards CD1d^+^ target cells and the highest IFNγ/IL-4 ratio. Future studies further assessing the anti-tumor function of CD8α iNKT cells would be of interest. We found similar *in vitro* killing of NALM cells by purified CD4^+^, CD4^-^, and bulk CD19 CAR-iNKT cells (unpublished study). *In vivo*, we observed similar anti-myeloma efficacy of BCMA CAR-iNKT cells that were either 41% CD4^+^ or 100% CD4^+^ (62) suggesting that CAR-iNKT function can occur across a range of CD4 expressing cells, though side by side comparisons of sorted cells *in vivo* could confirm this. Of high interest to the field is the discovery that CD62L, a memory marker, may be key to improved CAR-iNKT function. Tian et al. (85) showed that CD62L^+^ CD19 CAR-iNKT cells had prolonged persistence and efficacy *in vivo* compared to CD62L^-^ CD19 CAR-iNKT cells and that expansion in media containing both IL-2 and IL-21 preserved the CD62L^+^ population (84). Incorporation of IL-21 into the CAR construct also improved retention of CD62L expression and CAR-iNKT cell persistence and efficacy, although whether this was due to having IL-21 in culture, *in vivo* or both is not clear (89). This result stems from their original observation that *ex vivo* expanded CD62L^+^ NKT cells expressed higher IL-21 Receptor (IL-21R) mRNA compared to CD62L^-^ NKT cells suggesting this subset may be uniquely sensitive to IL-21. IL-2/IL-21 provided a survival effect via downregulation of the pro-apoptotic protein, BIM, which they posit contributes to improved anti-tumor function through a survival benefit to iNKT cells. Additionally, a recent study by a separate group demonstrated that when iNKT cells are transduced with a lentivirus expressing both B7H3-CAR and IL-21 (“armored iNKT”) tumor killing and *in vivo* persistence were increased while expression of exhaustion markers was decreased compared to iNKT cells transduced without IL-21 (89). However, IL-21 is known to facilitate interactions between CD1d^+^ B-cells presenting lipid antigens and iNKT cells in natural immune settings suggesting this might be a contributing factor. This may be independent of the CD1d-iNKT TCR interaction however, since Daudi cells are reported to be resistant to killing by the iNKT TCR (99, 100). One study reports Daudi cells are CD1d positive but not bound to B2 microglobulin (99) and in another study they are reported to be CD1d negative (100).

Building on the above studies, cytokines have also been employed to enhance CAR-iNKT cell expansion and function. Our group showed that BCMA CAR-iNKT cell expansion, persistence and anti-myeloma efficacy was enhanced when mice were treated with a long-acting IL-7 molecule (62). Similar results were shown for CAR-T cells due to decreased apoptosis and increased cell division (101), and our group is testing long acting IL-7 in relapsed/refractory large B cell lymphoma patients treated with CD19 CAR-T cells (NCT05075603). Results from this trial may support IL-7 treatment in patients receiving CAR-iNKT cells. Addition of human IL-15 cytokine to a GD2 CAR construct enhanced survival, efficacy, and trafficking of CAR-iNKT cells to tumor sites compared to a GD2 CAR without IL-15 (87). The GD2-IL-15 CAR construct is used in the only CAR-iNKT cell clinical trial with published results (82, 102) discussed further below. It remains to be seen which cytokine and delivery approach is safest and most effective. Addition of a cytokine to the CAR construct delivers local cytokine, although there is less control of cytokine levels as CAR-iNKT cells expand. Systemic treatment provides more control (i.e. treatment can be stopped) but with potential risk of unwanted systemic effects. Since IL-15 and IL-7 improve immune reconstitution after lymphodepletion, they may induce unwanted rejection of donor cells, as was shown in a trial employing memory-like natural killer cell therapy and IL-15 (103). Additional allogeneic pre-clinical studies along with current and future clinical trials are needed to clarify this.

### CAR-iNKT cells in the clinic

Currently, allogeneic CD19 CAR-iNKT cell therapy with a CD28 costimulatory domain, expressing secreted IL-15 and small hairpin RNAs targeting β2M and CD74 to downregulate HLA class I and II, respectively, are being tested in patients with B cell malignancies (ANCHOR/NCT03774654 and ANCHOR2/NCT05487651). Data from this trial presented at the Transplantation and Cellular Therapy Meeting on nine patients (81) showed one patient had a grade 1 CRS and three CRs and a PR were attained. Other ongoing trials include testing of CD19-IL15 CAR-iNKT cells (autologous) in patients with acute lymphoblastic leukemia, B-cell lymphoma, or chronic lymphocytic leukemia (NCT04814004) and a CD70 CAR-NKT cell clinical is enrolling patients with renal cell carcinoma (NCT06182735).

The only CAR-iNKT cell clinical trial to date with published data is the ongoing study (NCT03294954) testing autologous GD2-IL-15 CAR-iNKT cells in pediatric patients with relapsed or refractory neuroblastoma (82, 102). Twelve patients were treated with GD2-IL-15 CAR-iNKT cells, with an overall response rate of 25% and one case of grade 2 CRS (82). Five patients had PD, four SD, and three PR after one dose. Four patients received a second dose of GD2-IL-15 CAR-iNKT cells. The clinical status stayed the same for one patient (PR-PR), one converted from a PR to a CR (PR-CR), and two had progression (SD-PD and PR-PD). Of note, the responders had a higher percentage of CD62L expressing CAR-iNKT cells in the infused product than non-responders, corresponding with earlier pre-clinical data. Comparisons of pre-injected CAR-iNKT cells versus *in vivo* expanded CAR-iNKT cells in preclinical models and in clinical trials will further clarify which subset(s) of iNKT cells have the highest persistence and function. Although this trial demonstrated safety, further study is needed to support efficacy, clarification of the optimal dosing regimen, and the best timing for this intervention in the course of disease treatment.

Finally, of interest to the CAR-iNKT field, the presence of certain bacteria within the gut microbiome prior to autologous T cell harvest for CAR-T cell production predicted positive patient clinical responses by CAR-T cell therapy (104). Further, this also predicted higher abundance of peripheral T cells (104). Given that iNKT cells recognize microbial derived lipid antigens, and that iNKT phenotype and function are affected by the microbiome (105, 106), similar results might be predicted with iNKT cells and warrant future investigation. Together, although there is relatively little clinical CAR-iNKT cell data to date, these cells hold high promise for future therapeutic use.

## Discussion

There are a number of challenges for clinical translation of iNKT cell therapies. One major challenge for adoptive transfer of iNKT cells, whether for GVHD suppression or targeting malignant disease, is the rarity of iNKT cells and the ability to acquire clinically significant numbers for adoptive transfer. Further, donor variability is also an issue for autologous productions (107). This is not insurmountable, however, as multiple studies have shown the ease of expanding iNKT cells *ex vivo* (40, 41, 43, 62, 82, 85), and alternative approaches such as the engineering of iNKT cells from HSCs provide further possible methods (42, 86, 108, 109). In one study, Li et al. (86) reported a 10-11 week production starting with human CD34^+^ cells collected from either CB or G-CSF mobilized human peripheral blood stem cells, using an artificial organoid/αGalCer culture system. Remarkably, one CB could yield 5x10^11^ iNKT cells (mostly CD4^-^), highlighting the maximal potential for allogeneic “off-the-shelf” iNKT cell production.

Another concern inherent to any cellular therapy is the possibility of rejection. For use as an acute GVHD-suppressive therapy, rejection is unlikely in the early post-transplant severely immunocompromised environment. Further, the initial immunological steps required to elicit acute GVHD occur very early post-transplant (38, 39), so long-term persistence may not be required. Preventing rejection of CAR-iNKT cells may also be at least temporarily addressed via lymphodepleting chemotherapy as is used for current CAR-T therapies. Alternatively, genetic modification to delete HLA I and II in iNKT cells is feasible (82, 86, 102). Deletion of HLA I/II removes the “self” signal, which could lead to NK mediated killing, and potentially require strategies to prevent this such as addition of NK inhibitory ligands to iNKT cells (ie. HLA-C, HLA-E or CD47) (60, 110). Although somewhat surprising, there remains the possibility that deletion of HLA I and II in iNKT cells may not be necessary. Two separate studies show that the expression levels of HLAI and HLAII molecules are lower in expanded iNKT cells compared to T cells (86, 111). An interesting hypothesis from Rotolo et al. suggests that given the iNKT cell capacity to suppress T cell proliferation and activation in allogeneic GVHD responses, iNKT cells may also suppress the T cell proliferation and activation required for their own rejection (111). In their canine model, when a “good donor” was used (gene expression patterns suggesting a central/effector memory profile, low expression of exhaustion genes, and high telomerase, correlating with increased proliferation and function *in vitro*), they showed unmodified donor iNKT cell persistence up to 78 days in immune competent MHC mismatched recipients, though expansion of iNKT cells from peripheral blood was required to detect donor cells (40). Importantly, this study demonstrated a lack of rejection even in the presence of an intact immune system. Further studies directly comparing HLAI/II edited vs unedited iNKT cells and testing different (or no) lymphodepletion strategies for CAR-iNKT therapy will elucidate the rejection potential.

**Figure 1:**
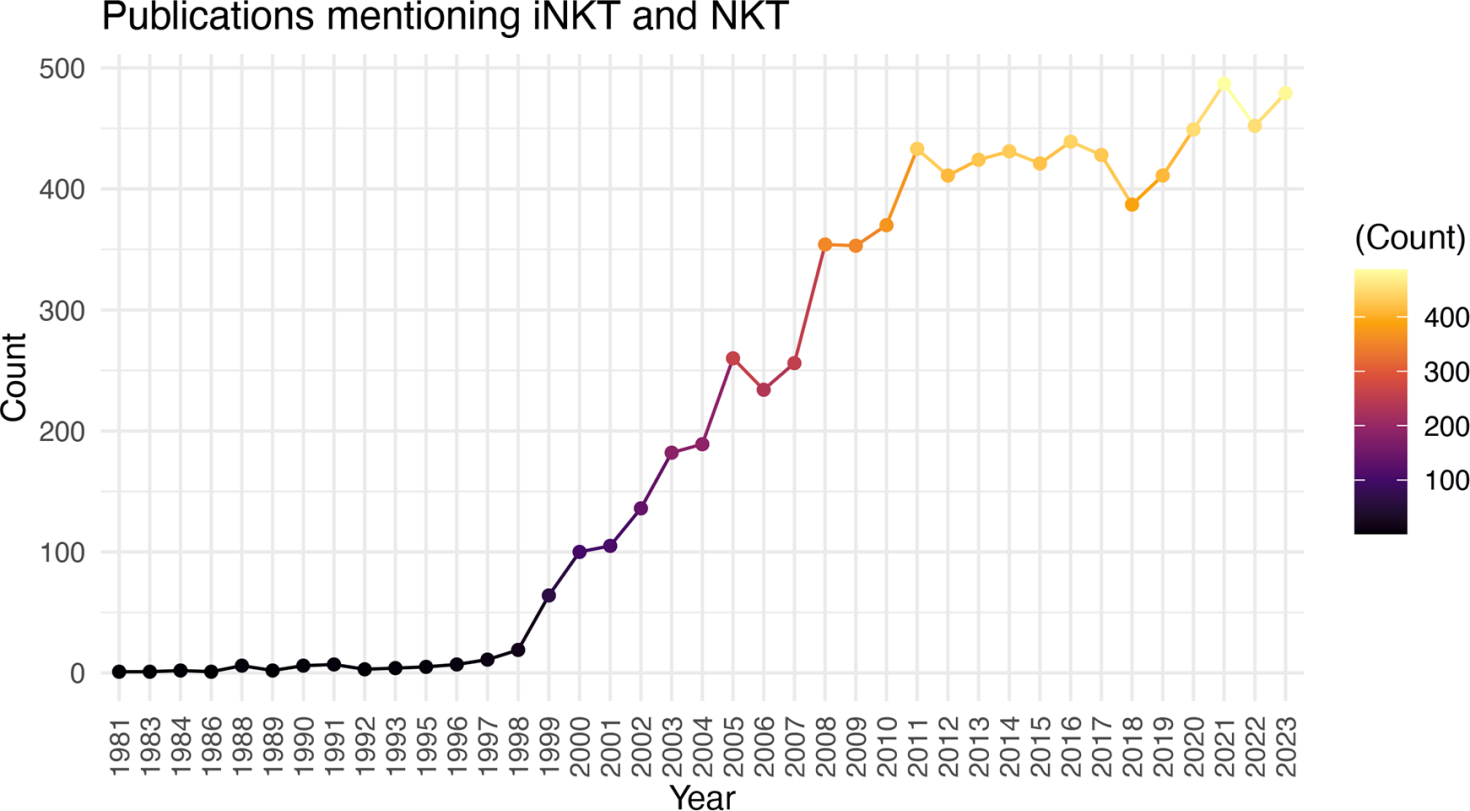
Increasing Number of iNKT Cell Publications Over Time. Number of publications (excluding reviews) by year using the search criteria “iNKT” and “NKT”.

There is significant promise for iNKT cell therapies, however, the field is still relatively young and there is much to learn about the biology, cellular subsets, production protocols, pre-treatment conditioning, and best strategies for editing iNKT cells when necessary (ie. to express CAR, delete HLA etc.). While it may appear as though the substantial preclinical promise of iNKT cell therapies is not resulting in tangible clinical translation, the significant time required from initial discovery to biologic understanding, to clinical trials, and finally to approved therapies is well known. The discovery of iNKT cells was well reviewed by Godfrey et al. (66), with the term Natural Killer T cells first used in 1995 (112) and an understanding of their CD1d restriction (2) and invariant TCR expression (1) around the same time. Further research into their activation, function, and other biologic features led to their first use as a therapeutic in preclinical models as early as 1999 (113). The first clinical trial to boost iNKT cell activation through αGalCer administration was in 2002 (114), followed by the first clinical trial of adoptive transfer in 2006 (73), and the first CAR-iNKT trial opening in 2018 (82, 102). Therefore, the field seems to be progressing on par with the development of other cell therapies. Accordingly, there has been a remarkable increase in the number of papers published on iNKT cells in the last 29 years (**Figure 1**). With time, there is no doubt the biology of these unique and powerful cells will be better understood, and their full translational potential realized.

## Author contributions

JO: Conceptualization, Writing – original draft, Writing – review & editing. MM: Conceptualization, Writing – original draft, Writing – review & editing. RJ: Data curation, Writing – original draft, Writing – review & editing. JD: Conceptualization, Writing – original draft, Writing – review & editing.
